# Association Between Anthropometry and Cognitive Impairment in Patients with Type 2 Diabetes: Secondary Analysis from the ‘Cognition in Diabetes’ Study

**DOI:** 10.17925/EE.2025.21.2.7

**Published:** 2025-10-27

**Authors:** Subhankar Chatterjee, Rana Bhattacharjee, Animesh Maiti, Moumita Mondal, Subir Hait, Souvik Dubey

**Affiliations:** 1. Department of Endocrinology & Metabolism, Medical College & Hospital, Kolkata, India; 2. Department of Neuromedicine, Bangur Institute of Neurosciences, IPGMER & SSKM Hospital, Kolkata, India

**Keywords:** Anthropometry, cognition, dementia, diabetes, lean diabetes, metabolic syndrome, mild cognitive impairment, obesity

## Abstract

With an ageing global population, the triad of type 2 diabetes (T2D), obesity and dementia poses a growing public health challenge. India harbours a notable proportion of younger patients with T2D with a non-obese or lean phenotype. However, data remain scarce on the impact of adiposity on cognition in T2D, particularly when assessed using a comprehensive, culturally and linguistically adaptable cognitive battery in a cohort free from major confounding factors. The aim of this study is to examine the relationship between various anthropometric indices and cognitive performance in patients with T2D. This cross-sectional observational study was conducted at the diabetic clinic of a tertiary hospital from 2022 to 2024. Eligible participants were patients with T2D aged 20–60 years with at least primary education. Exclusion criteria included non-T2D diagnosis, inability to communicate in Bengali and conditions known to impair cognition. A total of 125 patients with T2D were recruited. Demographics, diabetes-related variables and anthropometric measurements were recorded. Cognitive function was assessed using the Bengali version of the Addenbrooke’s Cognitive Examination (ACE-III). Statistical analysis was performed using Jeffreys’ Amazing Statistics Program (v.0.19). ACE-III total scores showed significant positive correlations with height, weight and neck circumference (NC) (p<0.001 for each). Attention was positively associated with height, weight, NC, neck–height ratio (NHR) and negatively with weight-adjusted waist index (WWI) (p<0.05 for all). Memory correlated positively with height and weight (p<0.05 for both). Language was positively related to height, weight and NC (p<0.05 for all) and negatively to WWI (p=0.011). Visuospatial ability positively correlated with height, weight, waist circumference (WC), hip circumference (HC), NC and NHR (p<0.05 for all). Lean patients with T2D had significantly lower visuospatial scores (p=0.040), lower ACE-III total scores (p=0.049) and a greater prevalence of cognitive impairment (p=0.032). In multiple linear regression, height (p=0.014) and HC (p=0.024) were independent predictors of ACE-III total score. This is the first Indian study to evaluate the association between anthropometric measures and cognition in T2D. Cognitive impairment and dementia were more prevalent in lean than in obese patients with T2D. Future studies incorporating imaging-based body composition analysis are warranted to identify modifiable anthropometric risk factors for cognitive decline in T2D.

The growing convergence of type 2 diabetes (T2D), obesity and dementia represents a pressing public health challenge in ageing populations worldwide.^1^ Robust epidemiological studies and meta-analyses have consistently shown that T2D is associated with accelerated cognitive decline.^1,2^ Beyond statistical correlation, a bidirectional relationship between T2D and Alzheimer’s disease has been proposed, as both conditions share overlapping pathogenetic mechanisms, including insulin resistance (IR), chronic inflammation and microvascular damage.^3^ T2D and its common comorbidities – dyslipidaemia and hypertension – are also established risk factors for vascular dementia.^4^

Obesity, a principal driver of IR and a major contributor to T2D, has likewise been linked to cognitive impairment.^5,6^ Yet, the relationship is complex: some studies, such as Natale et al., suggest that late-life obesity may confer partial protection against dementia and dementia-related mortality.^7^ This paradox underscores the multifaceted interplay among these three epidemics, which are connected by shared biological pathways including IR, systemic inflammation, atherosclerosis and arterial stiffness.^8^ Metabolic syndrome, the clinical manifestation of underlying IR, has also been implicated in cognitive decline, although results have been inconsistent.^9^ The coexistence of diabetes and obesity – termed ‘diabesity’ – would logically be expected to magnify dementia risk. However, evidence is mixed, with some studies reporting that obesity may even protect cognitive function in individuals with T2D.^10–12^ Such conflicting data may reflect the influence of unaccounted confounders, including age, educational attainment, cerebrovascular disease, hypoglycaemic events and psychiatric comorbidities, which can significantly bias observed associations.^13^ This highlights the importance of studying well-defined T2D cohorts in which alternative causes of cognitive impairment are rigorously excluded.

India, often described as the diabetic capital of the world, presents a unique context in which these relationships between diabetes, obesity and dementia can be explored. While obesity is rising, a substantial subset – up to 25% in certain reports – of individuals with T2D has a lean phenotype.^14^ Prior Indian research with stringent exclusion criteria has examined cognitive outcomes in T2D but has not systematically evaluated anthropometric measures.^15^ In older Indian adults, higher body mass index (BMI) has been linked to better cognitive performance, whereas underweight status has been associated with impairment, a relationship further modulated by physical strength indicators such as hand grip.^16^ A recent study has indicated that BMI<23 kg/m^2^ is significantly associated with cognitive impairment.^17^ Thus, it is imperative to clarify the complex role of body composition in the cognitive health of individuals with T2D and inform tailored intervention strategies.

Given these gaps, the current study was undertaken among patients with T2D to systematically examine the association between multiple anthropometric indicators of adiposity and cognitive function by employing a culturally and linguistically validated cognitive assessment battery within a rigorously selected cohort, controlling for known confounders.

## Materials and methods

The present study was part of the project titled CID (Cognition In Diabetes) and a secondary analysis of the T2D cohort of the parent study.^17^ This cross-sectional observational study was conducted at the Diabetic Clinic, Department of Endocrinology, Medical College & Hospital, Kolkata, between September 2022 and September 2024. Individuals with T2D, aged 20–60 years, who had completed at least primary education (up to class IV) and spoke Bengali as their primary language were considered eligible for inclusion.

Patients were excluded if they had any type of diabetes other than T2D (including secondary diabetes mellitus) or had a history of level 3 hypoglycaemia. Other exclusion criteria included chronic liver disease, stage IV or V chronic kidney disease, pregnancy, malignancy, vitamin B12 deficiency or any clinical condition requiring hospitalization.

Participants with diagnosed neuropsychiatric conditions (regardless of current psychotropic medication use), a history of central nervous system disorders (such as stroke, epilepsy, demyelinating diseases, space-occupying lesions or neuroimaging-confirmed abnormalities) or previously diagnosed neurodegenerative dementias were also excluded. Additional exclusions included comorbidities known to impair cognitive function (e.g. hyperthyroidism and uncontrolled hypothyroidism) and any sensory or physical impairments, such as significant visual, auditory or speech deficits, or motor dysfunction of the dominant hand, that could interfere with the reliable administration of cognitive assessments.

As described in the parent study, convenience systematic non-probability sampling was adopted.^17^ Every 10th patient presented to the diabetic clinic was evaluated for suitability to be included in the present study based on the above-mentioned inclusion and exclusion criteria. Thus, over the period of 18 months of data collection, 125 patients with T2D fulfilling both the inclusion and exclusion criteria were finally recruited. The statistical power of the study was calculated post hoc.

After obtaining the approval of the Institutional Ethics Committee of Medical College & Hospital, Kolkata (Ref No. MC/KOL/IEC/NON-SPON/1747/01/2023 dated 02 January 2023) and getting informed written consent in the local vernacular language (Bengali) from each patient, data collection was started with maintenance of anonymity. The research was conducted in accordance with the principles outlined in the Helsinki Declaration of 1964 and its later amendments.

Details about age, sex, educational status, socioeconomic status (according to the modified Kuppuswamy’s socioeconomic status scale), diabetes duration and blood pressure (and data about anti-hypertensive drugs) were taken.^18^ Data about fasting plasma glucose, 2 h post-prandial plasma glucose (by colourimetric enzyme method using Sclavo Konelab 600i Prime analyser), glycated haemoglobin (by high-performance liquid chromatography method using Biorad D10 HbA1c [glycated haemoglobin] analyser) (National Glycohemoglobin Standardization Program and Diabetes Control and Complications Trial-compliant) and fasting lipid profile (total cholesterol, high-density lipoprotein cholesterol, low-density lipoprotein cholesterol and triglycerides) by Cobas-Mira Roche autoanalyser machine with enzymatic techniques were noted. All the above investigations were done within one week before the cognitive evaluation. Capillary blood glucose was measured by glucometer (Accu-Chek^®^ Active [Roche Diagnostics GmbH, Mannheim, Germany]) just before the cognitive evaluation.

### Anthropometric measurements

- Weight: Measured with light clothing to the nearest 0.1 kg by a digital weighing machine (Tantia, Japan, Model-HA521, Lot number-860525) by the same observer, and an average of two measurements was taken.
- Height: Measured to the nearest 0.1 cm with a Charder HM200PW wall-mounted stadiometer (calibrated using a 36" calibration rod [Perspective Enterprise, Portage, Michigan, USA]) without shoes, with head in Frankfurt plane by the same observer, and an average of two measurements was taken.
- BMI: Calculated as weight in kg/(height in metres)^2^. Patients with a BMI ≤23 kg/m^2^ were categorized as having lean T2D.^14^
- For other anthropometric measurements, such as neck, waist and hip circumferences (HCs), a flexible, non-stretchable measuring tape was used.
- Waist or abdominal circumference (WC): Measured midway between the inferior border of the lowest rib and the superior border of the iliac crest, in the horizontal plane, at the end of normal expiration, with the patient standing and feet 23–30 cm apart.^19,20^ The measurement was recorded to the nearest 0.1 cm, performed by the same observer, and the average of two readings was taken.
  ○ A WC>90 cm for males and >80 cm for females was used as the cut-off to define obesity.^21^
  ○ For the diagnosis of metabolic syndrome, the WC cut-off values were >102 cm for males and >88 cm for females.^22,23^
- HC: Measured at the level of maximum lateral extension (the most prominent point) of the hips.^24^ Measurements were taken by the same observer, and the average of two readings was recorded to the nearest 0.1 cm.
- Waist–hip ratio (WHR): Calculated as WC (in cm) divided by HC (in cm). A WHR>0.9 for males and >0.8 for females was used as the cut-off to define obesity.^21^
- Weight-adjusted waist index (WWI): Calculated as WC (in cm) divided by the square root of weight (in kg). WWI, a relatively newer index of adiposity, has been negatively correlated with cognition, cardiovascular health, muscle mass and overall survival.^25–28^
- Neck circumference (NC): Measured at the level of the mid-neck (defined as the plane between the mid-cervical spine and mid-anterior neck), just below the laryngeal prominence, with the head positioned in the Frankfurt plane.^20^ Measurements were taken twice by a single observer, and the average was recorded to the nearest 0.1 cm. An NC>34 cm for males and >30.5 cm for females was used as the cut-off.^29^
- Neck–height ratio (NHR): Defined as NC (in cm) divided by the patient’s height (in metres), representing an adjusted NC to account for height-related variations.^20^ The same study has shown that NHR is a more reliable and reproducible marker of upper body obesity than NC alone. To the best of our knowledge, no published cut-off values exist for identifying upper body adiposity among Indians. Therefore, the reference values proposed by Selvan et al. (NHR>21.17 for males and >20.48 for females) as predictors of metabolic syndrome were adopted for the current study.

Patients were further stratified into the presence or absence of metabolic syndrome according to the National Cholesterol Education Program (NCEP) Adult Treatment Panel III (ATP III) criteria.^22,23^

### Cognitive evaluation

A detailed cognitive evaluation was conducted using the Bengali adaptation of the Addenbrooke’s Cognitive Examination-III (ACE-III).^30^ Despite its inherent limitations, ACE-III remains one of the most comprehensive and widely used screening tools for the diagnosis of mild cognitive impairment (MCI) and dementia, in both clinical and research settings.^31^ Notably, it has been adapted and validated in multiple Indian languages, making it a highly suitable instrument for comprehensive cognitive assessment in diverse linguistic populations.^30,32^

ACE-III evaluates five distinct cognitive domains: attention (maximum score: 18), memory (26), fluency (14), language (26) and visuospatial abilities (16), yielding a total maximum score of 100. As performance on ACE-III is significantly influenced by educational attainment, different diagnostic cut-offs have been established based on years of formal education. For individuals with formal education up to the 10th grade or higher, total scores below 85 and 88 were used as cut-offs for diagnosing dementia and MCI, respectively. In contrast, for those with less than the 10th-grade education, scores below 83 and 86 were used to diagnose dementia and MCI, respectively.^30^

### Statistical analyses

Statistical analyses were performed using the Jeffreys’ Amazing Statistics Program (JASP), version 0.19 (2024, Netherlands), MedCalc^®^ Statistical Software, version 23.0.2 (MedCalc Software Ltd., Ostend, Belgium), Python’s statsmodels.stats.multitest.multipletests (pvals, alpha=0.05, method='fdr_bh') and G*Power, version 3.1.9.6. A p-value of <0.05 was considered statistically significant.

The normality of continuous variables was assessed using the Shapiro–Wilk test, with p<0.05 indicating a non-parametric distribution. Continuous parametric variables were expressed as mean ± standard deviation (SD), while non-parametric variables were reported as median with interquartile range (IQR). Categorical variables were presented as absolute numbers and percentages.

For intergroup comparisons of categorical variables, Pearson’s chi-squared test was used, with Yates’ correction applied when appropriate. The chi-squared test for trend (Cochran–Armitage test) was used for ordinal categorical variables. When continuous variables were non-normally distributed, the Mann–Whitney U test was employed. Spearman’s rank correlation analysis was used to compare multiple groups of ordinal variables.

To assess linear associations between two continuous variables, Spearman’s rank correlation coefficient (Rho, r) with a 95% confidence interval (CI) was calculated, and scatter plots were examined. Simple linear regression, followed by multiple linear regression analysis, was performed where applicable. To address the risk of type I error due to multiple comparisons, the False Discovery Rate (FDR) was controlled using the Benjamini–Hochberg procedure. Adjusted p-values (q-values) were considered significant at q<0.05.

## Results

The baseline characteristics of the study sample are presented in *Table 1*. Among the participants, 28.8% were classified as lean diabetics (BMI <23 kg/m²), and 57.6% met the NCEP-ATP III criteria for metabolic syndrome. While 16% had MCI, 43.2% were diagnosed with dementia.

The ACE-III total score correlated positively with height and weight, but not with BMI. NC was also positively correlated with ACE-III total score; however, this correlation became non-significant after adjusting NC for height (NHR).

In subdomain analysis, attention was significantly and positively correlated with height, weight, WC, HC, NC and NHR. Visuospatial ability showed significant positive correlations with height, weight, BMI, WC, HC, NC and NHR. Memory was positively correlated with height and weight, but not BMI. Similarly, language function showed significant positive correlations with height, weight and NC. The WWI showed a negative correlation in the attention, fluency and language subdomains (*Table 2*).

**Table 1:** Baseline characteristics (n=125)

Variables			Value
Age (median ± IQR)			47 +/-15 years
Sex	Male		50.4%
	Female		49.6%
Education (median ± IQR)			9 +/-5
Socioeconomic status	Lower		0%
	Lower middle		9.6%
	Middle		47.2%
	Upper middle		36%
	Upper		7.2%
Duration of diabetes (median ± IQR)			8 +/-9 years
BMI <23 kg/m^2^			28.8%
Capillary blood glucose (median ± IQR)			179 +/-108 mg/dL
Fasting plasma glucose (median ± IQR)			120 +/-64 mg/dL
Post-prandial plasma glucose (median ± IQR)			176 +/-88.75 mg/dL
HbA1c (median ± IQR)			7.30 +/-2.30
Hypertension			39.2%
LDL-C (median ± IQR)			85 +/-50 mg/dL
HDL-C (median ± IQR)			42 +/-10 mg/dL
Triglycerides (median ± IQR)			134 +/-95 mg/dL
Metabolic syndrome			57.60%
ACE-III total score (median ± IQR)			85 +/-11
Cognitive state		Normal	40.80%
		MCI	16%
		Dementia	43.20%

*ACE-III = Addenbrooke’s Cognitive Examination-III; BMI = body mass index;*

*HbA1c = glycated haemoglobin; HDL-C = high-density lipoprotein cholesterol; IQR = interquartile range; LDL-C = low-density lipoprotein cholesterol.*

Other assessed variables, including age, duration of diabetes, hypertension, dyslipidaemia, metabolic syndrome, addiction and presence of micro- or macrovascular complications, were not significantly associated with total ACE-III score. In contrast, male sex (p=0.010), higher education (p<0.001, *r*=0.612) and higher socioeconomic status (p<0.001) were associated with higher ACE-III scores. After FDR adjustment, 23 of the 26 originally significant correlations (p<0.05) remained significant, including positive correlations of height, weight and NC with attention, visuospatial and total ACE-III scores and a negative correlation of WWI with language (*Table 2*).

Among anthropometric variables, NC <340 mm for males and <305 mm for females was significantly associated with cognitive impairment (p=0.036) (*Table 3*).

When comparing lean and non-lean patients with T2D, there were no significant differences in age, education status, duration of diabetes, HbA1c and lipid profile. However, non-lean T2D was significantly associated with female sex, higher socioeconomic status, hypertension and metabolic syndrome. Importantly, lean T2D had significantly lesser visuospatial ability (p=0.040), lesser ACE-III total score (p=0.049) and more cognitive impairment (p=0.032) (*Table 4*).

A multiple linear regression analysis was conducted to examine the relationship between the ACE-III total score and three predictor variables identified through simple linear regression: height, HC and NC. The regression model was statistically significant, F(3, 121)=6.834, p<0.001, indicating that the predictors collectively accounted for a substantial portion of the variance in ACE-III total score. Specifically, the model explained 14.5% of the variance in the outcome variable. Height (coefficient [b]=0.285, standard error [SE]=0.114, p=0.014, 95% CI: 0.059, 0.511) and HC (b=0.230, SE=0.101, p=0.024, 95% CI: 0.030, 0.431) emerged as significant predictors of ACE-III total score. In contrast, NC was not a significant predictor (p=0.073). Collinearity diagnostics indicated no evidence of severe multicollinearity. After controlling for the other predictors, both height and HC showed moderate associations with ACE-III total score. These relationships remained consistent after adjusting for the most recent HbA1c, education and socioeconomic status. When analysed by gender, height and HC were significant predictors of ACE-III total score in males. However, in females, none of the anthropometric measures were significantly associated with the score.

**Table 2:** Relationship between various anthropometric parameters and Addenbrooke’s Cognitive Examination-III cognitive scores*

	p Value (Spearman’s rho coefficient)					
	Domain score					
Variables	Attention	Memory	Fluency	Language	Visuospatial	Total score
Height	<0.001* (0.395)	0.011* (0.227)	0.080	0.002* (0.274)	0.012* (0.223)	<0.001* (0.314)
Weight	<0.001* (0.362)	0.008* (0.237)	0.074	0.011* (0.226)	<0.001* (0.339)	<0.001* (0.298)
BMI	0.329	0.322	0.739	0.895	0.018*^,†^ (0.212)	0.230
Hip circumference	0.035*^,†^ (0.189)	0.153	0.164	0.985	0.001* (0.285)	0.052
Waist circumference	0.049*^,†^ (0.176)	0.229	0.993	0.835	0.011* (0.227)	0.107
Waist–hip ratio	0.319	0.421	0.311	0.879	0.457	0.464
Weight-adjusted waist index	0.044* (-0.181)	0.318	0.047* (-0.178)	0.011* (-0.227)	0.336	0.073
Neck circumference	<0.001* (0.424)	0.101	0.091	0.025* (0.201)	0.002* (0.280)	<0.001* (0.292)
Neck–height ratio	0.023* (0.203)	0.883	0.243	0.471	0.026* (0.200)	0.128

*Spearman’s correlation statistics was performed. Strength of correlation has been mentioned as Spearman’s rho coefficient value in parenthesis if the correlation is found to be statistically significant (p<0.05).

*^†^Parameters that lost significance following False Discovery Rate (FDR) adjustments.*

*BMI = body mass index.*

**Table 3:** Association of anthropometric parameters with cognitive status

		Cognitive status according to ACE-III			
Variables		Normal (%)	MCI (%)	Dementia (%)	p
BMI (kg/m^2^)	Underweight (n=3)	0	0	100	0.284^†^
	Normal (n=33)	33.33	9.09	57.58	
	Overweight (n=36)	55.56	16.67	27.78	
	Obese (n=53)	37.74	20.76	41.51	
Waist circumference (cm)	>90 cm for males or, >80 cm for females (n=101)	43.56	15.84	40.59	0.402^‡^
	≤90 for males or, ≤80 for females (n=24)	29.17	16.67	54.17	
Waist–hip ratio (cm/cm)	>0.90 for males or, >0.85 for females (n=116)	40.52	17.24	42.24	0.382^‡^
	≤0.90 for males or, ≤0.85 for females (n=9)	44.44	0	55.56	
Neck circumference (mm)	≥340 cm for males or, ≥305 cm for females (n=112)	43.56	15.84	40.59	0.036*^,‡^
	<340 cm for males or, <305 for females (n=13)	29.17	16.67	54.17	
Neck–height ratio (cm/m)	>21.17 for males or, >20.48 for females (n=96)	40.63	19.79	39.58	0.083^§^
	≤21.17 for male or, ≤20.48 for females (n=29)	41.38	3.45	55.17	
Metabolic syndrome	Yes (n=72)	43.06	15.28	41.67	0.835^§^
	No (n=53)	37.74	16.98	45.28	

*p<0.05 had been reported as significant.

*^†^Spearman’s rank correlation statistics.*

*^‡^Chi-square test with Yates’ correction.*

*^§^Chi-square test.*

*ACE-III = Addenbrooke’s Cognitive Examination-III; BMI = body mass index; MCI = mild cognitive impairment.*

Post hoc power analysis was conducted to evaluate the achieved power for the linear regression model with ACE-III total score as the dependent variable and height, HC and NC as predictors. Based on the observed effect size f^2^=0.00192, residual variance=75.697 and R^2^=0.145, the analysis achieved a power of 0.95 at an alpha level of 0.05.

## Discussion

Although numerous studies have shown associations between diabetes and dementia, and between obesity and cognitive impairment, there is limited research on the effects of obesity or leanness on cognition in T2D. This study addresses this gap by evaluating the relationship between multiple anthropometric measures and cognitive performance in younger individuals with T2D.

In this study, the total cognitive score was significantly positively correlated with height, weight and NC. Subdomain analysis showed that attention positively correlated with height, weight, WC, HC, NC and NHR, but negatively with WWI. Memory was positively correlated with height and weight. Language was positively associated with height, weight and NC, but negatively with WWI. Visuospatial ability was positively correlated with height, weight, BMI, WC, HC, NC and NHR. Overall, lean T2D demonstrated significantly greater cognitive impairment.

Evidence from studies involving infants, children and adolescents suggests that cognitive abilities are influenced by the pace of physical development.^33–35^ Greater adult height may be correlated with a higher cognitive reserve, which could contribute to the preservation of cognitive function in later life. Indeed, height has been identified as an independent and strong predictor – alongside age and education – of cognitive performance.^36^ Final adult height may serve as a proxy for multiple early-life factors such as childhood illnesses, access to quality education, socioeconomic status and overall health – all known to influence cognitive outcomes.^36,37^ Whether a common genetic predisposition contributes to both taller stature and enhanced cognitive ability remains a subject of ongoing investigation.^36,38^ Neuroimaging studies suggest that variations in brain volume and surface area may underlie the observed association between height and cognition.^39,40^ The relationship between height and cognitive function in individuals with diabetes was first examined by West et al., who reported that shorter stature was associated with cognitive deficits in males but not in females.^41^ However, contrasting findings were observed in a separate study, where short stature was also linked to cognitive impairment among diabetic Nigerian females compared with controls.^42^

In the present study, higher body weight was associated with better cognitive performance. This observation may be explained by the fact that weight loss can precede the onset of cognitive impairment.^43–45^ A study by Ding et al. reported that elderly patients with T2D with cognitive impairment experienced weight loss more frequently than those with preserved cognition.^46^ However, an earlier meta-analysis by Wang et al. did not find any significant association between weight loss and cognitive status in individuals with T2D.^47^ A more accurate understanding of the complex relationship between weight and cognition in diabetes would require longitudinal data capturing both pre- and post-morbid BMI. We did not observe a significant association between BMI and the total ACE-III score. Subdomain analysis revealed a positive correlation between BMI and visuospatial ability; however, it lost statistical significance after FDR adjustment. The existing literature on BMI and dementia risk remains inconsistent. A prospective study conducted in the UK found that individuals with a BMI>30 kg/m² had a 35% increased risk of developing dementia after a decade-long follow-up compared to those with a normal BMI. Notably, this association remained significant even after adjusting for potential confounders.^5^ Similarly, Mallorquí-Bagué et al. analysed the execute function of older individuals with overweight/obesity and metabolic syndrome participating in the PREDIMED-PLUS study and showed that BMI had a direct negative effect on executive function among patients with T2D.^48^ Conversely, a large retrospective UK cohort study showed that being underweight during middle and older age was linked to a higher risk of dementia over the following two decades, and the incidence of dementia decreased with increasing BMI category.^49^ A more recent study by Li et al. sought to clarify this association using 40 years of follow-up data. Their findings indicated that individuals whose BMI increased during early mid-life and subsequently declined in later mid-life had a higher risk of developing dementia compared with other BMI trajectory groups.^50^ In contrast, a similar study by Russ et al. found no association between BMI in early life and dementia risk five decades later. Interestingly, they observed that individuals who experienced BMI decline in their 50s had a higher risk of dementia-related mortality compared with those whose BMI declined in their 70s.^51^ Additionally, another study by Li et al. suggested that maintaining BMI within a specific range could serve as a protective factor for cognition in individuals with T2D.^12^ Supporting this, a post hoc analysis from the Action to Control Cardiovascular Risk in Diabetes–Memory in Diabetes (ACCORD-MIND) trial proposed that obesity might be protective for cognitive function in T2D, potentially mediated by greater total brain volume observed in obese individuals.^10^

**Table 4:** Comparison between lean type 2 diabetes and non-lean type 2 diabetes (n=125)

Variables		Lean T2D (BMI ≤23 kg/m^2)^) n=36	Non-lean T2D (BMI >23 kg/m^2^) n=89	p
Age (median ± IQR)	43.50 +/-11.75 years	48 +/-16 years	0.378^†^
Sex	Male	19.20%	31.20%	0.021*^,‡^
	Female	9.60%	40%	
Education (median ± IQR)		8 +/-6 years	9 +/-3 years	0.198^†^
Socioeconomic status	Lower	0%	0%	0.012*^,§^
	Lower middle	4.80%	4.80%	
	Middle	16.80%	30.40%	
	Upper middle	5.60%	30.40%	
	Upper	1.60%	5.60%	
Duration of diabetes (median ± IQR)		10 +/-9 years	7 +/-9 years	0.212^†^
HbA1c (median ± IQR)		7.60 +/-3.90%	7.10 +/-2.10%	0.136^†^
LDL-C (median ± IQR)		82.50 +/-55.50 mg/dL	85 +/-47 mg/dL	0.889^†^
HDL-C (median ± IQR)		43 +/-9 mg/dL	42 +/-12 mg/dL	0.789^†^
Triglycerides (median ± IQR)		128.50 +/-100.25 mg/dL	134 +/-83 mg/dL	0.723^†^
Hypertension		5.60%	33.60%	0.004*^,‡^
Metabolic syndrome		10.40%	47.20%	0.002*^,‡^
ACE-III attention (median ± IQR)		16.50 +/-2	17 +/-3	0.087^†^
ACE-III memory (median ± IQR)		19 +/-6.25	20 +/-6	0.072^†^
ACE-III fluency (median ± IQR)		10 +/-2.25	11 +/-2	0.273^†^
ACE-III language (median ± IQR)		25 +/-2	25 +/-2	0.740^†^
ACE-III visuospatial (median ± IQR)		13 +/-2	14 +/-4	0.040*^,†^
ACE-III total score (median ± IQR)		83 +/-13.50	86 +/-10	0.049*^,†^
Diagnosis	Normal	8.80%	32%	0.032*^,§^
	MCI	2.40%	13.60%	
	Dementia	17.60%	25.60%	

*p<0.05 was taken as statistically significant.

*^†^Mann–Whitney U test.*

*^‡^Chi-square test.*

*^§^Chi-square test with Yates’ correction.*

*ACI-III = Addenbrooke’s Cognitive Examination-III; BMI = body mass index; HbA1c = glycated haemoglobin; HDL-C = high-density lipoprotein cholesterol; IQR = interquartile range; LDL-C = low-density lipoprotein cholesterol; MCI = mild cognitive impairment; T2D = type 2 diabetes.*

HC, often considered a protective adiposity index against various metabolic disorders, including T2D, has also been associated with better cognitive outcomes.^52^ Findings from the present study echoed this association. Although HC was not significantly correlated with the composite ACE-III score, it showed significant positive correlations with attention and visuospatial abilities. However, following FDR adjustment, the association of HC with attention lost its statistical significance. A study conducted among a Taiwanese population found no significant association between HC and cognitive performance as measured by the Mini-Mental State Examination (MMSE).^53^ In contrast to the majority of existing literature, WC in the present study demonstrated a weak yet significant positive correlation with attention (lost significance post FDR adjustment) and visuospatial domains.^12,53–55^ Nonetheless, WWI – a more robust marker of central adiposity than WC alone – was negatively correlated with attention, fluency and language domains, although not with the total ACE-III score.^25^ These findings align with recent studies by Huang et al. and Wang et al., both of which reported negative correlations between WWI and cognitive performance.^26,56^ The association between WHR and cognition has been reported as negative in some studies, although others have not found such a relationship.^54,57–60^ In the present study, no significant association was observed between WHR and cognitive function.

NC, a useful marker of upper body adiposity and internal carotid artery intimal thickness, has been found to be negatively correlated with cognitive performance among elderly Chinese individuals.^61^ Shin et al. observed an inverse association between NC and grey matter volume, which became particularly pronounced in the presence of diabetes.^62^ However, no such association was observed in a study conducted among the US population.^63^ The significant positive correlation between NC and cognitive scores found in our study was unexpected and warrants further validation through large-scale, longitudinal, multi-ethnic studies. Notably, this association lost significance after adjusting for height. It is important to note that NC is influenced not only by neck adipose tissue but also by the girth of several neck muscles.^64^ Interestingly, neck muscle cross-sectional area has been positively associated with brain volume in healthy older men.^65^ Additionally, NC has been negatively correlated with declines in activities of daily living, frailty and undernutrition in the elderly.^66,67^

It is intriguing to explore why some obesity parameters have been associated with better cognitive performance in this study, as well as in several previous studies.^10–12^ Some researchers have identified weight loss as a prodromal symptom occurring well before the clinical onset of dementia.^43–45^ In this context, the theory of reverse causation has been proposed, suggesting that obesity may appear to be protective against dementia.^68^ While Xing et al. reported an association between greater total brain volume and better cognitive function in obese individuals with T2D, most studies have observed the opposite trend.^10,69–71^ Elevated levels of oestrogen and leptin have also been proposed as plausible explanations for preserved cognition among elderly individuals with obesity.^60,72,73^ On the contrary, Zhai et al. have suggested a role of low energy expenditure as an independent risk factor of cognitive impairment among middle-aged and elderly patients with T2D.^74^

The present study had several limitations. It was a single-centre study with a relatively smaller sample size, and underweight and normalweight subjects were probably under-represented. Moreover, we did not analyse the physical activity levels of the participants. A longitudinal study design would enhance the understanding of the cause-and-effect relationship between obesity and cognition in T2D. The true relationship between adiposity, lean body mass and cognitive impairment in diabetes would be better judged by studies specifically focusing on detailed body composition analysis rather than relying solely on anthropometric measures.^75,76^

However, this study is a significant addition to the existing literature for several reasons. First, we selected patients with T2D after excluding all possible confounders, such as lack of formal education, advanced age-related cognitive decline, any evidence from neuroimaging or clinical history suggestive of coexisting brain diseases, or known psychiatric disorders. Second, we employed the best available culturally and linguistically adaptable comprehensive cognitive battery, enabling robust assessment across all cognitive domains. Finally, we evaluated multiple clinically practical obesity-related parameters, all of which demonstrated consistent correlations, thereby enhancing the reliability of our findings.

## Conclusion

Lean T2D is associated with greater cognitive impairment compared with its overweight or obese counterpart. However, in the present study, BMI is not significantly correlated with the ACE-III total score. Certain adiposity indices showed positive correlations with better cognitive performance, with height and HC emerging as significant predictors of cognitive impairment among young and middle-aged patients with T2D. Further large-scale studies incorporating the imaging techniques to assess the body composition are needed to identify modifiable anthropometric risk factors for cognitive impairment. In the absence of disease-modifying pharmacotherapy to halt the progression of dementia, such findings could be particularly valuable for long-term management, helping to preserve independence and quality of life among patients living with diabetes.
